# Immunoglobulin light chain amyloidosis diagnosis and treatment algorithm 2018

**DOI:** 10.1038/s41408-018-0080-9

**Published:** 2018-05-23

**Authors:** Morie A. Gertz

**Affiliations:** Mayo Clinic, SW Division of Hematology, 200 First Street, Rochester, MN 55905 USA

## Abstract

Immunoglobulin light chain amyloidosis (AL) should be considered in any patient that presents to a cancer care provider with nephrotic range proteinuria, heart failure with preserved ejection fraction, non-diabetic peripheral neuropathy, unexplained hepatomegaly or diarrhea. More importantly, patients being monitored for smoldering multiple myeloma and a monoclonal gammopathy of undetermined significance (MGUS) are at risk for developing AL amyloidosis. MGUS and myeloma patients that have atypical features, including unexplained weight loss; lower extremity edema, early satiety, and dyspnea on exertion should be considered at risk for light chain amyloidosis. Overlooking the diagnosis of light chain amyloidosis leading to therapy delay is common, and it represents an error of diagnostic consideration. Algorithms will be provided on how to evaluate patients with suspected AL amyloid as well as how to manage patients referred from other medical specialties with biopsy-proven amyloid. An organized stepwise approach to the treatment of patients with light chain amyloidosis, including established and investigational therapies, will be reviewed.

## Patient 1

A 78-year-old female was found to have an IgA Κ monoclonal protein in April of 2016. Her hemoglobin was 16.8. The M spike was 1.3 g/dL. Her IgA was 1960 mg/dL, *κ* free light chain 4.57 mg/dL, *λ* 1.32 mg/dL, ratio 3.46. She was reassured that this was a monoclonal gammopathy of undetermined significance (MGUS) with no evidence of multiple myeloma. One year later, she was seen at the Mayo Clinic because of a progressive decline. Her weight had fallen from 68 to 48 kg. She complained of numbness in her feet. She had multiple syncopal episodes and intractable diarrhea. Her blood pressure was 94/64. This constellation of weight loss, neuropathy, orthostatic hypotension, and diarrhea led to a bone marrow biopsy, a fat aspirate, and a lip biopsy, all of which demonstrated amyloid deposits. Due to her frail state, melphalan and dexamethasone were recommended. She died three months later. Comment: This would be a typical patient being monitored with MGUS for the development of multiple myeloma when she had AL amyloid for a year before treatment was initiated. By the time the diagnosis was established, the disease was advanced and intervention was unlikely to provide benefit.

## Introduction

The incidence of AL amyloidosis is estimated to be three to five patients per million per year^1^. This statistic would make it approximately one-fifth as common as multiple myeloma^2^. In the United Kingdom, the incidence is ~1 per 100,000^3^. The Medicare claims database suggests that the mean age of AL amyloidosis at diagnosis is 63 with an incidence of 10–14 patients per million per year with a prevalence higher in males^4^. It is estimated that there are 12,000 adults in the United States currently living with AL amyloidosis. Wild-type TTR may be present in a quarter of the elderly at post mortem and is seen in 13–19% of patients with heart failure and preserved ejection fraction, likely making it the most common form of systemic amyloidosis^5^.

The diagnosis of AL amyloidosis should be considered by a cancer care provider in any patient seen with nephrotic range proteinuria, heart failure with preserved ejection fraction^6^, non-diabetic peripheral neuropathy^7^, unexplained hepatomegaly^8^, or diarrhea. This is much easier to list than it is to recognize in practice. Heart failure with preserved ejection fraction, one of the most common manifestations of AL amyloidosis, can be misdiagnosed because the echocardiogram has nonspecific findings. Wall thickening can be misinterpreted as hypertension with hypertrophy or hypertrophic cardiomyopathy^9^. Although cardiac magnetic resonance imaging with gadolinium can be quite specific, this test is often not ordered unless the diagnosis is suspected^10^. A pseudoinfarction pattern seen on the EKG could be misinterpreted as true ischemic disease. Patients with peripheral neuropathy and a monoclonal gammopathy are frequently misdiagnosed as CIDP (chronic inflammatory demyelinating polyneuropathy)^11^. These patients can undergo months of immunoglobulin infusions or plasma exchange before a diagnostic evaluation for AL amyloidosis is initiated. Monitoring for the physical signs of AL amyloidosis, such as tongue enlargement or periorbital purpura, is not adequate as these are found in only 15% of patients. Although these findings are highly specific for AL amyloidosis, they are very insensitive and their absence should never be used to exclude a diagnosis of AL amyloidosis.

For the cancer provider following patients with MGUS or smoldering multiple myeloma, it is important to keep in mind that these patients are not monitored solely for the development of myeloma. Some develop lymphoma or Waldenström macroglobulinemia, and a small percentage develop light chain amyloidosis^12^. At Mayo Clinic, 9% of all patients seen with a monoclonal gammopathy are ultimately proven to have light chain amyloidosis. Even adjusting for referral bias, 3–4% of all patients with monoclonal proteins seen have light chain amyloid. Furthermore, if a provider does not see one patient with AL amyloidosis for every five patients with multiple myeloma, it is likely the diagnosis is being overlooked^13^.

Evidence that delays in diagnosis have not, as yet, been overcome is reflected by early mortality statistics for newly diagnosed AL amyloidosis patients at Mayo Clinic^14^. Nearly 20% of patients succumb to the disease within 6 months of diagnosis, and this statistic has shown no improvement in 40 years, suggesting, as in patient 1, that patients who are diagnosed at an advanced state cannot be helped despite major advances in therapy for this disease.

An online survey from the Amyloid Research Consortium indicates that 37% of patients are diagnosed over one year from the onset of initial symptoms with a median of three physician visits before a diagnosis is established. Cancer care providers constitute 34% of the specialists that are diagnosing this disorder, far greater than nephrologists, cardiologists, and gastroenterologists^15^.

**Fig. 1:**
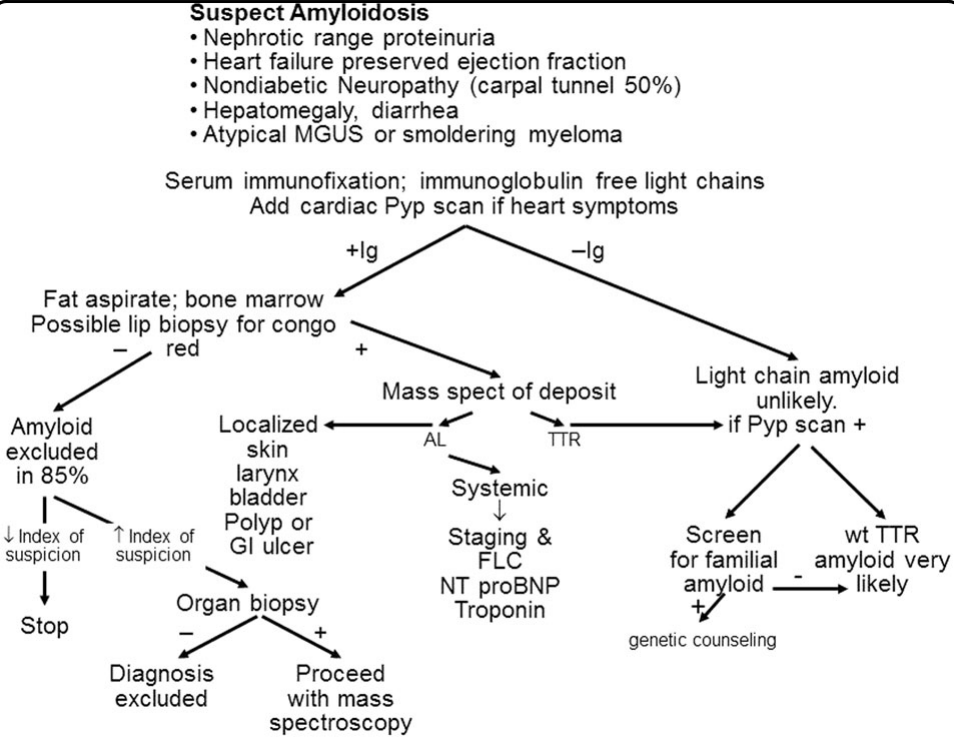
Diagnostic algorithm for patients that are being evaluated for a syndrome compatible with systemic amyloidosis

If AL amyloidosis is suspected, particularly in patients who have multi-organ dysfunction, biopsies are not the first step in screening. Currently, 71% of patients that are seen have cardiac involvement, 58% have renal involvement, 23% have nerve involvement, and 16% have liver involvement. Despite these numbers, the majority of patients with cardiac, renal, hepatic, and nerve problems will not have AL amyloidosis. The first screening test for these patients, as shown in an algorithm (Fig. 1), would be serum immunofixation and an immunoglobulin free light chain assay for k and λ immunoglobulin light chains^16^ and, if the patient has cardiac dysfunction, a pyrophosphate scan^17^, which should be available in virtually every hospital in the United States. If these screening tests are positive, the diagnostic pathway is clear. For patients with immunoglobulin light chain abnormalities, doing a simple subcutaneous fat aspirate (https://www.youtube.com/watch?v=tctYTmxd9gQ) and a bone marrow biopsy will demonstrate amyloid in over 85% of patients. Only if the index of suspicion is high would the patient next move to direct organ biopsy of the heart, liver, kidney, nerve, etc. Patients with echocardiographic evidence suggesting cardiac amyloidosis, usually demonstrating thickened ventricular walls, Doppler evidence of poor diastolic filling and abnormal longitudinal strain need to undergo a pyrophosphate scan which if positive should be considered TTR and not light chain amyloidosis. These patients should be referred to a cardiologist for tissue sampling to validate amyloidosis.

### Classification of amyloid

Accurate typing of the protein subunit responsible for amyloid deposition is important since it directs treatment. Only systemic immunoglobulin light chain amyloidosis is treated with chemotherapy or stem cell transplantation. In all other forms of amyloid, be it systemic or localized, chemotherapy is contraindicated. Historically, we used immunohistochemistry^18,19^ and immunofluorescence^20^ to classify the amyloid. These techniques have major drawbacks. In light chain amyloidosis, commercial antisera purchased to detect κ and λ immunoglobulin light chains are usually directed against epitopes on the constant region of the immunoglobulin light chain. When amyloid light chains are deposited in tissues, usually only a fragment of the intact light chain is deposited, typically the variable portion (V_L_) with a molecular weight of ~12 kd. A normal intact light chain has a molecular weight of 25 kD, suggesting there has been deletion of the constant portion of the light chain, making the immunoglobulin fragment unrecognizable to commercial antisera. Secondly, the light chains of amyloid are known to misfold, and the potential of a previously exposed epitope no longer being accessible to the commercial antibody exists, rendering it unidentifiable^21^. Finally, there are at least 30 different types of amyloid proteins, and few centers are equipped with such a large panel of antisera, making it next to impossible to identify rare forms of amyloidosis, such as fibrinogen, LECT2, apolipoprotein, and lysozyme.

The gold standard for typing is laser capture mass spectroscopic proteome analysis^22^. In this technique, amyloid deposits are directly removed from a glass slide, and the technique can be performed on archived paraffin-embedded tissues. Peptides are sequenced by a mass spectrometer and then compared with libraries of proteins for identification. In addition to specifically identifying the subunit protein, the technique serves as a positive control since it will also detect associated proteins always seen in amyloid deposits, such as serum amyloid P, vitronectin, and apolipoprotein E^23^. Although expensive and not available in all laboratories, proteomic analysis with mass spectroscopy remains the gold standard for identification of the amyloid protein subunit.

In a review of over 4000 proteomic analyses of amyloid deposits, 62% were of immunoglobulin origin. However, a full 38% were not of immunoglobulin origin, and chemotherapy would have been contraindicated. Non-immunoglobulin forms of amyloidosis included AA amyloid, ALECT2, A-insulin, A-fibrinogen, and A-gelsolin (Kurtin P. personal communication).

## Patient 2

The patient was a 71-year-old male diagnosed with renal AL amyloid nephrotic syndrome in March of 2007 and underwent a stem cell transplant in December of 2007, obtained an organ response with urinary protein falling from 10.5 g/24 h to 1.5 g/24 h. The patient developed congestive heart failure in July of 2013 and underwent an endomyocardial biopsy that demonstrated amyloid. Proteomic typing demonstrated that this patient had TTR cardiac amyloid, and genetic studies demonstrated the *TTR* gene to be wild-type, so-called senile cardiac amyloidosis. He died at the age of 79 of progressive heart failure 39 months following the diagnosis of TTR cardiac amyloidosis. Comment: This patient had two types of amyloidosis. He had AL amyloidosis successfully treated and had not relapsed after 9.5 years but subsequently developed age-related cardiac amyloidosis that could have easily been misdiagnosed as relapsing AL if cardiac biopsy and proteomic analysis had not been done.

Over 15 years ago, it was common to identify amyloid in a patient with a monoclonal gammopathy and assume that this was AL type. However, in 81 patients with TTR amyloidosis, an M protein was found in 20 of the 81 and an abnormal free light chain ratio in 8 of the 81^24^. A second study of wild-type TTR amyloidosis also demonstrated a monoclonal protein in 25% of patients^25^. Finally, even when AL is diagnosed with proteomic analysis, this does not indicate whether the amyloidosis is localized or systemic. Attention must be given, particularly to those patients who present with amyloid in a skin biopsy, bladder biopsy, laryngeal biopsy, or at the edge of a colonic ulcer or polyp, that the amyloid may be a localized AL amyloidosis that requires no intervention^26^.

### Patient is referred from a specialist with biopsy-proven amyloid

**Fig. 2:**
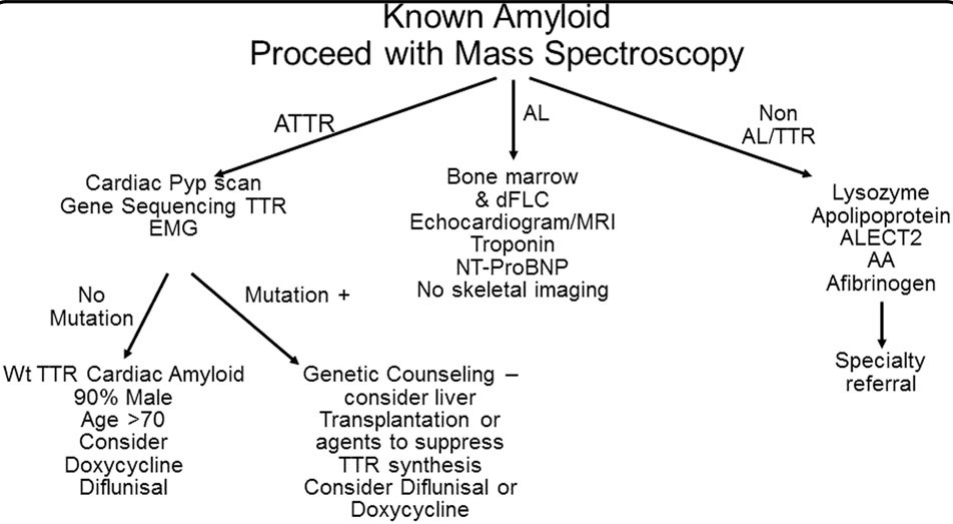
Diagnostic algorithm for a patient referred with an established tissue biopsy diagnosis of amyloidosis

Many specialists, when encountering a patient with biopsy of an organ containing amyloid, refer to a cancer care provider uncertain of the type of amyloidosis. The first step for all biopsied tissues, shown in an algorithm (Fig. 2), would be mass spectroscopic analysis. In patients with AL amyloidosis, measurement of bone marrow plasma cells^27^ and FISH genetics^28^, as would be done in multiple myeloma patients, are indicated. For staging purposes, one needs to know the NT-proBNP, troponin, and the difference between the involved and uninvolved immunoglobulin free light chain^16^. If not already done, echocardiography or magnetic resonance imaging of the heart is required since the extent of cardiac involvement is important for prognosis^29^. For patients with light chain amyloidosis in the absence of symptoms, the role of routine skeletal imaging, as is done in multiple myeloma, is not well defined due to a lack of high quality evidence.

**Fig. 3:**
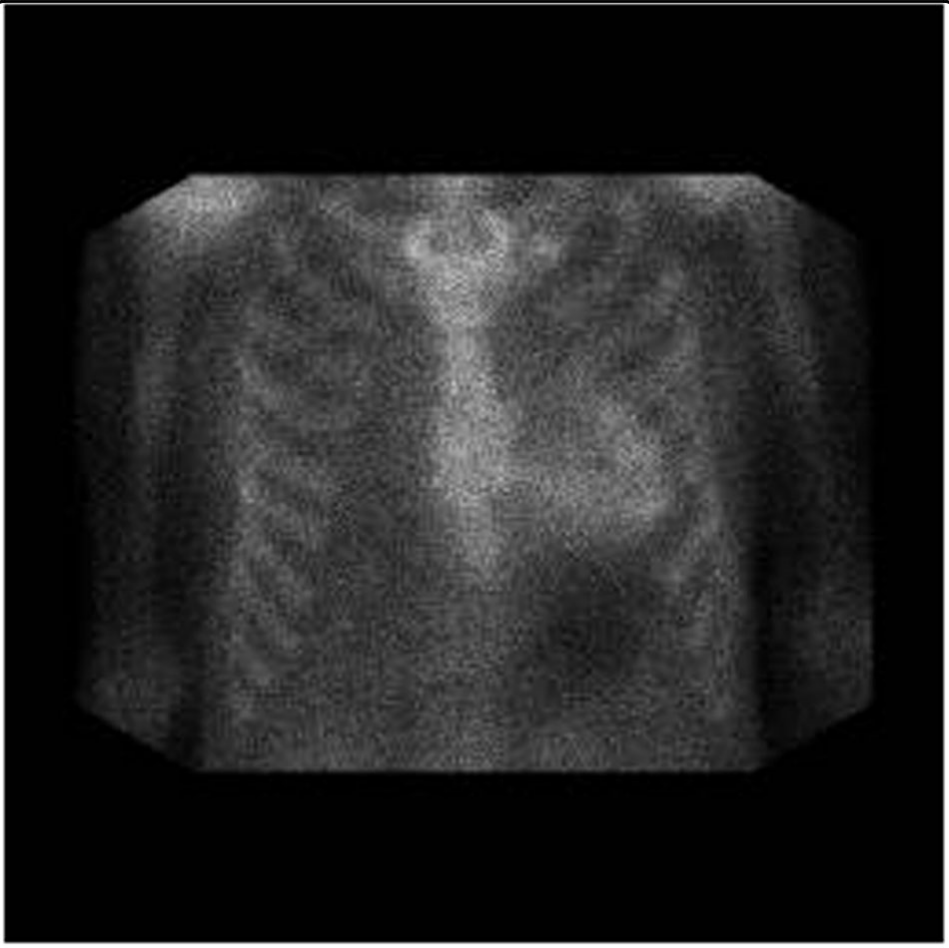
Pyrophosphate scan of a patient with TTR cardiac amyloidosis

If ATTR is identified by mass spectroscopic analysis, this patient should have presented with peripheral neuropathy or cardiomyopathy. The next step in evaluation would be pyrophosphate scanning of the heart (Fig. 3). A strong positive scan would suggest that the amyloid is of TTR origin^30^. Any patient with TTR amyloid should have gene sequencing of the *TTR* gene to distinguish wild-type TTR, as is seen in senile cardiac amyloidosis, from the very rare mutations of TTR that lead to inherited amyloidosis^31^. Since familial amyloidosis is not treated with chemotherapy, these patients should be referred for genetic counseling, consideration of liver transplant, diflunisal^32,33^ or doxycycline therapy^34^, or one of the expanded access programs for agents that suppress translation of liver TTR messenger RNA^35,36^ into the fully-formed TTR protein. Patients with wild-type TTR amyloidosis are usually over the age of 70, 90% are men, and half have carpal tunnel syndrome^37^. Currently, there is no standard of therapy. Although the evidence is weak, trials of diflunisal and doxycycline should be considered (rationale discussed below).

Staging of AL amyloidosis is based on a four-point system where one point is assigned for a DFLC > 18 mg/dL, a cardiac troponin T > 0.025 mcg/L, or an NT-proBNP ≥ 1800 ng/L. This provides a staging system of I, II, III, IV based on the number of points assigned (0, 1, 2 or 3). The staging system has been validated in multiple datasets, including patients treated with stem cell transplantation, patients on clinical trials, and non-transplant patients treated with standard chemotherapy^38^. Other effective staging systems include a European staging system where Mayo 2004 stage 3 was sub-classified into 3 sub-stages using systolic blood pressure and NT-proBNP at 100 mm Hg^39^ and 8500 ng/mL^40^, respectively and a model based on the number of involved organs, creating a 4-stage model (1 organ, 2 organs, 3 organs, 4 or more organs; organ model).

#### Therapy of amyloidosis

**Fig. 4:**
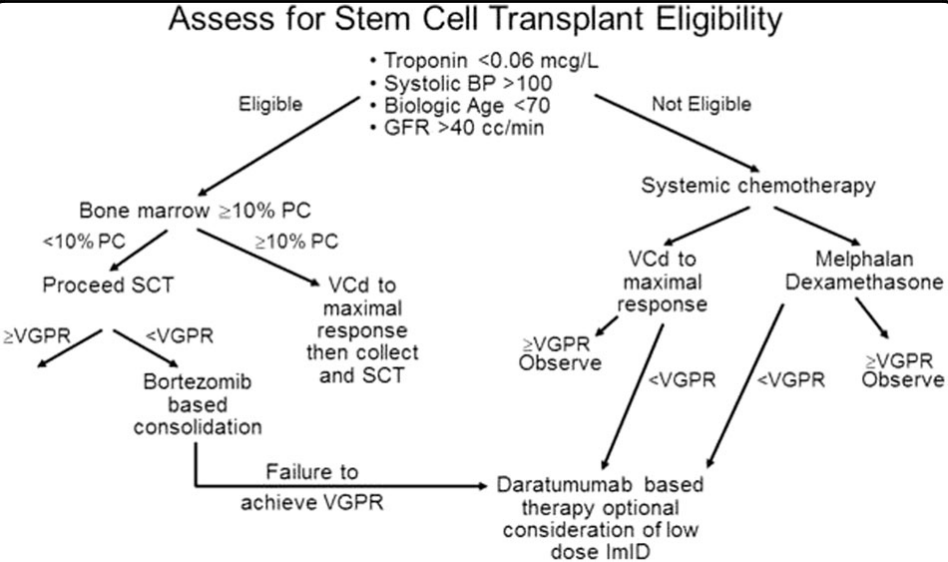
Current treatment algorithm in light chain amyloidosis

The first successful treatment for AL amyloidosis was melphalan and prednisone introduced in 1972^41^. Autologous stem cell transplantation was reported in 1996^42^. High-dose dexamethasone was introduced in 1997^43^. Melphalan and dexamethasone was reported in 2004^44^. There have been multiple reports on the use of thalidomide^45,46^, lenalidomide^47,48^, and pomalidomide^49,50^, as well as combinations of IMIDs with alkylating agents^51^, but IMIDs are poorly tolerated in patients, particularly those with cardiac AL amyloidosis^52,53^. The first step in assessing therapy for an AL amyloid patient, as shown in an algorithm (Fig. 4), is determination of their eligibility for stem cell transplantation. Using transplantation in AL amyloid is theoretically better than it is for multiple myeloma. Unlike multiple myeloma, the tumor mass being treated is less with a median of approximately 10% plasma cells at diagnosis and a median dFLC of only 24 mg/dL. Unfavorable genetics, seen in nearly a quarter of patients with multiple myeloma [such as 1q+, t(4;14), and −17p] are present in <5% of patients with light chain amyloidosis. The proliferative rate of plasma cells is lower in AL amyloidosis patients, suggesting that once a response is obtained, it is likely to be more durable than is seen in multiple myeloma^54^. In fact, in the pre-novel agent era, ten-year survival of patients with AL amyloidosis undergoing stem cell transplantation was 43%^55^. A prospective randomized trial of melphalan and dexamethasone with stem cell transplant also favored stem cell transplantation, although the comparator arm did not contain novel agents^56^. With careful patient selection, the therapy-related mortality has been reduced to approximately 2%^57^. Patients that do not achieve greater than a VGPR can have bortezomib-based consolidation post-transplant, which significantly upgrades treatment response post-transplant^58^. A prospective randomized trial demonstrated an improved survival outcome with bortezomib-dexamethasone prior to stem cell transplant^59^. The current policy at Mayo Clinic is to give induction chemotherapy for patients who have >10% plasma cells prior to proceeding to stem cell transplant (Fig. 4).

Even with the strong preference for autologous stem cell transplant, no more than 25% of newly diagnosed patients are eligible by virtue of age, renal function, and extent of cardiac failure. The remaining 75–80% are candidates for chemotherapy. Melphalan and dexamethasone demonstrates impressive survival in patients that are capable of receiving full-dose therapy with a median survival of just less than 8 years^60^. There have been reports of cyclophosphamide-thalidomide-dexamethasone^61^, lenalidomide-dexamethasone, melphalan-dexamethasone-lenalidomide, cyclophosphamide-lenalidomide-dexamethasone^62^, but none of these are currently used in the Mayo Clinic algorithm due to toxicity and the preference for bortezomib. It should be noted that lenalidomide raises the NT-proBNP in AL patients^63^. CyBorD or VCD (cyclophosphamide-bortezomib-dexamethasone) was first reported to be effective in 2012. In the original iteration, cyclophosphamide was given orally weekly, dexamethasone orally weekly, and bortezomib subcutaneously weekly. In this original trial, 17 patients were treated, 10 with symptomatic cardiac involvement with a 94% response rate and 71% complete response rate with an additional 3 patients who were previously deemed ineligible for stem cell transplant to become eligible^64^. These results were validated in over 230 patients with AL amyloidosis, demonstrating a median survival in excess of six years, with all patients surviving in stage 1 disease and a median survival of less than one year in stage 4 disease^40^. Survival was dependent on response depth, with patients achieving a VGPR or better having the best outcome. Achievement of a VGPR is used in the algorithm to determine whether second-line therapy should be considered. In using bortezomib-based therapy, one needs to be aware that response rate is poor in patients with t(11;14)^65^, a genetic abnormality seen in nearly 50% of patients with AL amyloidosis. The presence of t(11;14) should lead one to strongly consider stem cell transplantation over bortezomib, since this genetic abnormality does not have an unfavorable impact in transplanted patients. Predictors of early death after therapy initiation include the Mayo stage and greater than two organs involved. The value of cyclophosphamide when combined with bortezomib remains unproven^66^.

Daratumumab, approved for the treatment of relapsed multiple myeloma as a single agent as well as in combination with lenalidomide or bortezomib, clearly shows activity in the treatment of patients with AL amyloidosis^67^ and appears to have a low-toxicity profile. In 2017, 24 patients with light chain amyloidosis were reported, and only 5 failed to achieve a PR or better; 9 of the 24 achieved a complete response^68^. ClinicalTrials.gov lists two phase 2 trials assessing daratumumab in the treatment of AL amyloidosis (NCT02841033 and NCT02816476). The combination of VCd and daratumumab is also recruiting as a phase 3 trial (NCT03201965).

### Venetoclax and new proteosome inhibitors

Because of the high prevalence of t(11;14) in AL amyloidosis patients, Venetoclax^69^, which has activity in multiple myeloma, particularly in those with the t(11;14), would be a natural candidate for the treatment of AL amyloidosis. It is given orally three days a week and does not appear to have cardiac toxicity^70^. There is a phase 1 trial underway in patients ClinicalTrials.gov (NCT03000660).

Carfilzomib, the second-generation proteasome inhibitor, has been tested^71^. A high incidence of cardiac involvement with AL amyloid makes it a challenging agent to use. Traditional pre- and post-hydration can aggravate patients predisposed to congestive heart failure. Carfilzomib is associated with cardiotoxicity in nearly 10% of patients. A review of Medicare admissions presented at the American Society of Hematology showed that carfilzomib-treated patients had a higher risk of hospitalization^72^. Hematologic responses have been reported, but its potential cardiotoxicity may be a barrier for wider implementation of this agent. Ixazomib has been the subject of a phase 2 trial with manageable toxicity and no cardiorespiratory toxicity (NCT01659658). A phase 3 trial of ixazomib-dexamethasone vs. physician-selected standard of care is underway (NCT01864018).

### Diflunisal and doxycycline

Diflunisal plays no role in the treatment of AL amyloidosis but may play a role in the treatment of wild-type and mutant TTR amyloidosis by preventing destabilization of the TTR tetramer^73^. A phase 3 trial demonstrated benefit in patients with mutant TTR neuropathy^74^. Given its efficacy, it is a consideration off label for patients with wild-type TTR amyloid and TTR cardiac amyloid.

Doxycycline has been used in patients with both AL^75^ and TTR^76–78^ amyloidosis with cardiac involvement. In vitro, doxycycline appears to disaggregate formed fibrils^79^. A trial from Mayo Clinic demonstrated that patients who achieved a hematologic response to stem cell transplant had a significantly longer overall survival post stem cell transplantation when given doxycycline compared to those receiving penicillin^80^. In a second study, which was case control, 26 patients receiving doxycycline were matched to 50 controls. The response rate was significantly higher in the doxycycline compared to controls, and the 12-month survival was 84 vs. 58%. Although there is no high-quality evidence and it has not been validated in a prospective randomized trial, doxycycline is a consideration if no other therapies are feasible.

### Monoclonal antibodies to dissolve amyloid

Although chemotherapy can effectively reduce the light chain burden and disrupt further deposition of AL amyloid, it does nothing for resident amyloid in tissues. Three monoclonal antibodies are undergoing studies now in patients with light chain amyloidosis that have derived maximal benefit from chemotherapy but have persistent organ dysfunction. The NEOD antibody was administered to a total of 69 patients. Among 14 cardiac evaluable, there were 8 responders. Among 15 renal evaluable, there were 9 responders^81,82^. The manufacturer discontinued the development of NEOD001 for AL Amyloidosis because the Phase 2b PRONTO study did not meet its primary or secondary endpoints. In addition the Phase 3 VITAL study was discontinued based on futility analysis. The murine monoclonal antibody, 11-1F4, recognizes an amyloid-associated conformational epitope^83^. In 26 patients, 8 were evaluable for organ response and 5 achieved this. No toxicity >grade 3 was recognized. This trial is ongoing^84,85^. The third antibody approach is targeting serum amyloid P component, which has the potential to disaggregate the amyloid fibril. Pretreatment with Miridesap depletes serum amyloid P so that the antibody dezamizumab can access amyloid in tissues^86^. This antibody may be applicable to all forms of amyloid, not just AL or TTR. It has been demonstrated to reduce the stiffness of the liver, and SAP scanning has shown regression of deposits^87^. Amyloid fibril targeted therapy with monoclonal antibodies is promising for the management of all forms of amyloidosis. Dissolution of amyloid fibrils can improve organ function.

### Organ transplantation

In AL amyloidosis, selected patients may successfully undergo renal or cardiac transplantation to assist with organ recovery. For patients that have single-organ involvement and control of the plasma cell proliferative process, organ transplantation may be considered. Stem cell transplantation can be safely performed in patients with dialysis-dependent^88^ renal failure^89^. Failure to achieve a complete response is no longer considered a contraindication to organ transplantation because of the increased availability of therapeutic options and direct organ donor programs. Once the patient has an established complete response, consideration of renal transplantation may be undertaken. Cardiac transplantation has also been performed in patients with AL amyloidosis^90^. However, most patients with advanced cardiac AL amyloidosis are not candidates for high-dose therapy and may tolerate standard-dose chemotherapy poorly. In these patients, it may be appropriate to do cardiac allografting and then follow with autologous stem cell transplantation^91–93^. Long-term survivorship has been reported in highly selected patients who fulfill the criteria of deep hematologic response and single-organ involvement^94^. Lenalidomide therapy is best avoided in organ transplant recipients that are considered for post organ transplant chemotherapy^95^.

## Conclusion

The most important first step is suspicion of the diagnosis of AL amyloidosis. That would allow earlier diagnosis when therapeutic intervention is most likely to be efficacious. Patients diagnosed with late cardiac disease will not benefit from any form of therapy due to their advanced degree of organ dysfunction. Once AL amyloid is suspected, the diagnosis can usually be made noninvasively, and deep organ biopsy is not generally required. Mass spectroscopic analysis should be standard for all newly diagnosed patients with amyloidosis to ensure correct classification of the protein subunit. All patients with light chain amyloidosis need cardiac biomarkers, free light chain measurements, and a bone marrow, with a thorough cardiac evaluation. The treatment of choice remains stem cell transplantation, but this is applicable to only the minority of patients. Patients who are candidates for stem cell transplant need evaluation for induction chemotherapy. For patients that are not transplant eligible, the standard of care for fit and unfit patients would be bortezomib-based. For very frail patients, all oral therapy with melphalan and dexamethasone is appropriate. Second-line therapy can include immunomodulatory-based therapies, but daratumumab seems to have a very high response rate and is likely to be used earlier in the disease. Anti-amyloid antibodies are likely to have a potential role in the future management of these patients.
